# 

**Published:** 1879-04

**Authors:** 


### CONTENTS.

COMMUNICATIONS.

Address. *By W. H. Morgan, D. D. S.* ........ 141
Impacts, and the Law that Governs Them. *By W. H. Robinson, D. D. S., Suisun, Cal* ........ 147
Where is the Fault. *By Dr. R. L. Cochran* ........ 155
Dental Nomenclature and Terminology. *By J. Taft* ........ 167
A Case in Practice ........ 170

PROCEEDINGS OF SOCIETIES.

Mississippi Valley Dental Society ........ 171

EDITORIAL.

Re-Union of the Alumni of the Ohio College of Dental Surgery ........ 175
Mal-Practice Case ........ 179
Dental Department of the University of Pennsylvania—Commencement ........ 180
Obituary ........ 181
For the Information of Dentists ........ 183
Mississippi Valley Dental Society ........ 183
A Desirable Result ........ 184

